# Correlates of Treatment and Disease Burden in People Living with HIV (PLHIV) in Italy

**DOI:** 10.3390/jcm11020471

**Published:** 2022-01-17

**Authors:** Antonella Cingolani, Alessandro Tavelli, Franco Maggiolo, Annalisa Perziano, Annalisa Saracino, Francesca Vichi, Massimo Cernuschi, Giovanni Guaraldi, Eugenia Quiros-Roldan, Antonella Castagna, Andrea Antinori, Antonella d’Arminio Monforte, on behalf of Icona Foundation Study Group

**Affiliations:** 1 Infectious Diseases Unit, Fondazione Policlinico Universitario A. Gemelli—Università Cattolica Del Sacro Cuore, 00168 Rome, Italy; 2 Icona Foundation, 20145 Milan, Italy; alessandro.tavelli@fondazioneicona.org; 3 Division of Infectious Diseases, ASST Papa Giovanni XXIII, 24127 Bergamo, Italy; franco31556@hotmail.com; 4 For CAB Icona Associazione Arcobaleno AIDS ODV, 10135 Torino, Italy; anna_perz_it@yahoo.it; 5 Clinic of Infectious Diseases, Department of Biomedical Sciences and Human Oncology, University of Bari “Aldo Moro”, 70124 Bari, Italy; annalisa.saracino@uniba.it; 6 Infectious Diseases Unit 1, Santa Maria Annunziata Hospital, Azienda USL Toscana Centro, 50012 Florence, Italy; francesca.vichi@uslcentro.toscana.it; 7 Department of Infectious Diseases, IRCCS San Raffaele Scientific Institute, University Vita-Salute San Raffaele, 20127 Milan, Italy; cernuschi.massimo@hsr.it (M.C.); castagna.antonella1@hsr.it (A.C.); 8 Department of Infectious Diseases, University Hospital of Modena, 41125 Modena, Italy; giovanni.guaraldi@unimore.it; 9 University Department of Infectious and Tropical Diseases, University of Brescia, ASST Spedali Civili, 25123 Brescia, Italy; eugeniaquiros@yahoo.it; 10 HIV/AIDS Department, INMI, L. Spallanzani, IRCCS, 00149 Rome, Italy; andrea.antinori@inmi.it; 11 Clinic of Infectious Diseases, Department of Health Sciences, University of Milan, ASST Santi Paolo e Carlo, 20142 Milan, Italy; antonella.darminio@unimi.it

**Keywords:** treatment burden, HIV, antiretroviral, long acting

## Abstract

Treatment burden is a multidimensional concept, including several aspects of life of patients affected by chronic conditions. It has been poorly explored in people living with HIV (PLHIV). An online anonymous survey of PLHIV taking antiretroviral therapy (ART) was conducted, in order to investigate the self-reported correlates of disease burden. HIV Treatment and Diseases Burden (TDB) was investigated with a questionnaire containing 31 items in 7 domains. Respondents were stratified in high burden (H-TDB)/low burden (L-TDB) according to overall HIV TDB mean + 1 standard deviation. Factors associated with H-TDB has been evaluated with a logistic regression model. In total, 531 PLHIV completed the questionnaire. 99 PLHIV had a H-TDB (18.6%). PLHIV with H-TDB were younger (*p* < 0.001), less frequently on current two drug antiretroviral (ARV) regimens (*p* = 0.01) and more frequently with plasma HIV-RNA >50 copies/mL (*p* = 0.04). At multivariable regression analysis, younger age (aOR 1.43, 95%CI 1.14–1.80; *p* = 0.002), not fully treatment satisfaction (aOR 2.19, 95%CI 1.28–3.74; *p* = 0.004), the need of a more accurate dialogue with treating physician (aOR 2.29, 95%CI 1.21–4.36, *p* = 0.01) and a self-declared lower overall Health Status (aOR 1.75, 95%CI 1.33–2.32; *p* = 0.002) were all associated with a H-TDB. One out of five PLHIV showed a high level of treatment and disease burden. Younger age, not fully satisfaction with ART and need of interaction with a tailored health system should be taken into consideration as correlates of treatment and disease burden in a patient-centered approach, to reduce the negative impact that it can produce on the overall perceived health status of the person.

## 1. Introduction

Forty years have passed since the first detection and isolation of the human immunodeficiency virus (HIV), but HIV still remains a global public health issue with 1.5 million of new HIV infection/year, a total of 37.7 million of people living with HIV (PLWH) in 2020 and 27.5 million of subjects on active antiretroviral therapy. The expanded and universal access to antiretroviral therapy (ART) together with a declining incidence of HIV, resulted in a significant decrease of the numbers of death from HIV/AIDS with a currently estimated 680,000 PLWH dying from HIV/AIDS globally in the year 2020 (64% fewer than in 2004 and 47% fewer than in 2010) [1].

The antiretroviral therapy has allowed to achieve substantial benefits over the years, in terms of prevention of new infections, life-expectancy and long-term management of the infection, but this should not cancel the actual difficulty of living with a chronic condition as HIV.

HIV- and ART-associated complications/issues emerged in the last two decades, resulting HIV in a novel chronic disease. Complications of residual inflammation or immunodeficiency such as cardiovascular diseases, cognitive disorders and cancers are rising in importance; moreover, the cumulative exposure to potentially toxic antiretroviral (ARV) drugs can led to clinically relevant disturbances (such as metabolic, liver, renal complications). This multimorbidity and related polypharmacy associated with HIV could deeply affect a healthy ageing for PLWH [2]. In addition to common aspects of other chronic diseases, HIV has important social, behavioral and stigma-related implications that can significantly impact the perceived and experienced burden by people living with HIV (PLHIV).

HIV infection, indeed, now represents a paradigm of chronic condition, in which the burden of both ‘disease’ and ‘treatment’ can significantly impact the patient’s life. The treatment burden can be defined as the impact of health care on patients’ functioning and well-being [3,4], considering every aspect of the patients’ health care life such as visits to the doctor, medical tests, treatment, treatment management, and lifestyle changes.

It has been demonstrated that treatment burden is associated, independently of illnesses, with adherence to care [5,6] and to hospitalization and survival rates [7,8].

One of the aspects to be considered, although not the only one, in the assessment of the treatment burden, is represented by the experience that the patient has with the medicines he/she takes daily. Positive experiences with medicines lead to improved control of patients’ symptoms or disease conditions and clinical markers, while negative experiences can manifest as adverse events (AE), poor disease control, inconvenience or inappropriate use of medicine. Nevertheless, patients’ experiences with medicines have been poorly considered [9,10,11,12,13,14,15] and very few efforts have been made to conceptualize treatment-related burden in chronic Illness [12,13,16,17,18,19,20,21].

Long-acting formulations can administer effective medications for months, releasing people from daily assumptions. The future availability of injecting long acting agents (iLAA) as antiretroviral regimen, could therefore, represents a potential revolution in terms of changing the treatment burden for PLWH and helping to manage the psychological burdens of HIV including non-desired disclosure of HIV status and preventing a constant reminder of an underlying disease [22].

The objective of this survey is to investigate the potential correlates of disease burden from the point of view of PLHIV and to try to correlate the disease burden with the overall self-reported health status. A focus on patients’ interest about Long-Acting Agents, the next frontier of antiretroviral therapy, is also briefly reported.

## 2. Materials and Methods

An online anonymous survey was conducted through the centers participating to the ICONA Study (Icona Network), with also the help of Italian Patient Advocacy Groups websites, between February and April 2021 (90 days).

The Icona Study is an Italian cohort of patients living with HIV, set-up in 1997 including HIV-1 infected subjects, naïve from ART at enrollment, further details of the study are described elsewhere [23]. All the 55 centers participating to Icona Study constitute the Icona Network, often involved for dissemination of surveys and other scientific projects of Icona among all the PLWH in care in each center.

Study data of the anonymous questionnaire has been collected using the online survey function of the RedCap electronic data capture tool hosted by Icona Foundation, through the website www.caricoditrattamento.it [24].

In the HIV-outpatient clinics of Icona Network, sites some brochures have been distributed, to inform the ART-experienced patients that might be involved in the survey, about the aims and the procedures of the study. Upon beginning the survey, participants were informed that the survey would take approximately 15 min and a brief informed consent form to study participation has been presented.

The HIV Treatment and Diseases Burden (TDB) has been investigated with a questionnaire adapted from the PETS questionnaire Patient Experience with Treatment and Self-Management by DT Eton et al., Qual Life Res 2017 [25] a comprehensive, patients-reported measure of the overall treatment burden. The general PETS questionnaire of DT Eton et al. has been re-focused only HIV-specific treatment and health related questions (Supplementary Materials). The questionnaire contains 31 items in seven domains: learning about health conditions and care (six items), medications (four items), difficulty with taking medications (two items), medical appointments (four items), interpersonal challenges (four items), limitations of role and social activity (6 items), and physical/mental exhaustion (five items). Items of the questionnaire use a 5-point ordered, categorical response Likert-scale depending on content domain (e.g., very easy to very difficult, not at all to very much, strongly agree to strongly disagree, never to always). 

Before the specific HIV TDB, the survey included other three sections collecting (i) socio-demographic variables, (ii) HIV-related data (date diagnosis, HIV markers, current ARV therapy, treatment satisfaction) and (iii) health status and behavioral data (Supplementary Materials).

Data were analyzed using descriptive statistics, median and interquartile range (IQR) for continuous, absolute and relative frequencies for categorical variables.

Respondents were stratified in high HIV Treatment and Diseases Burden (H-TDB) if they scored above the overall (on 31 items) HIV TDB mean + 1 standard deviation (SD), and with low HIV Treatment and Diseases Burden (L-TDB) if they scored below this threshold (2.94 points). Chi-square and Kruskall–Wallis tests have been used to compare characteristics of the two groups.

Factors associated with H-TDB were evaluated with crude and adjusted logistic regression models. The following response/variables has been included in the multivariable model: age, current antiretroviral (ARV) regimen, current reported HIV-RNA, treatment satisfaction, interaction with physician and overall health status.

Through one of the questions of the survey, in the second section on HIV data (“I would…to be able to take my HIV treatment not every day, thanks to specially designed medications”), it was also possible to understand the proportion and the characteristics of patients interested in the new non-daily formulations of ARV (injecting Long-Acting agents—iLAA). Chi-square test has been used to compare clinical and demographics characteristics between those subjects interested or not interested in iLAA. The results of the overall HIV TDB and of the seven domains of the TDB have been compared in the two groups using weighted mean and linear regression models with weights (as not all the questions are completed for all the participants in the HIV TDB questionnaire, the number of respondents for each item has been used as weight). The comparison for each single item of the TDB has been conducted with arithmetic mean and *t*-test. 

## 3. Results

### 3.1. General Characteristics of Respondents

The questionnaire was completed by 531 PLHIV. General characteristics of the participants are reported in Table 1. In total, 87% were male, 93% had Italian nationality, 42% had a University degree level of education, median age was 49 years (IQR 39–56), 60% declared a stable employment, 61% were Men who have sex with other Men (MSM), 88% declared current undetectable HIV-RNA and 57% CD4 cell count >500/mmc, 51% of respondent were diagnosed as PLHIV after 2010. Regarding antiretroviral treatment, 58% of PLHIV started antiretroviral therapy after 2010, 64% declared to assume an ARV regimen containing three drugs, while 31% contained two drugs. A current Single Tablet Regimen (STR) was reported in 74% of respondents. 52% of PLHIV also reported to be affected by comorbidities and 17.5% reported to assume more than five pills/day. 

### 3.2. Treatment Satisfaction and Relationship with Physician

In total, 51% of PLHIV reported to be fully satisfied with current ARV regimen, 45% reported a good but “not fully satisfaction” with ARV and 4% reported to be unsatisfied at all with ARV treatment. The desire to take an ARV regimen other than on a daily basis was reported in 57% of respondents. 

In total, 38% would like to be informed by their physician on new drugs available, 10% of PLHIV would be more involved in ARV decisions and 30% would require a deeper dialogue with treating physician. 

### 3.3. High Treatment and Disease Burden Definition and Proportions According with Self-Reported Data

The mean TDB was 2.18 (SD = 0.76). Based on the assumption defined in the Method section, patients with high TDB were defined as those with >2.94. 99 PLHIV had a H-TDB (18.6%), 432 PLHIV had a L-TDB (81.3%). 

PLHIV with H-TDB were younger (44 vs. 50 years *p* <0.001), less frequently assuming 2 ARV drug regimens (21 vs. 143; *p* = 0.01) and more frequently with reported plasma HIV-RNA >50 copies/mL (8 vs. 19; *p* = 0.04) (Table 1).

### 3.4. Factors Associated with H-TDB at Univariate and Multivariable Regression Analysis 

Factors associated with H-TDB were reported in Table 2. Younger age, the use of current ARV with 3 ARV drugs, the current reported HIV-RNA, the lack of fully satisfaction with current ARV, the need of stricter relationship with treating physician, and a low overall health were reported as associated with a H-TDB at univariate analysis. Nevertheless, in the adjusted model only younger age (per 10 years younger aOR 1.43, 95%CI 1.14–1.80; *p* = 0.002), not fully treatment satisfaction (vs. fully satisfaction aOR 2.39, 95%CI 1.40–4.09; *p* = 0.001), the need of a more accurate dialogue with treating physician (aOR 2.75, 95%CI 1.32–5.05, *p* = 0.001) and a lower overall self-declared Health Status (per 1 point lower, aOR 1.75, 95%CI 1.32–2.32; *p* < 0.001) were all independently associated with a H-TDB. The presence of other comorbidities, as well as the daily dosing frequency and the total number of pills/day taken were not associated with high HIV Treatment and diseases burden (Table 1). 

### 3.5. High Treatment and Disease Burden and Correlation with Quality of Life

PLHIV reported 2 (1–3) as median (IQR) score of Physical Health, 2 (2–3) for Mental Health, 3 (3–5) for Sexual health and 2 (2–3) for Overall Health, defined as overall median of all domains. Overall Health showed a OR of 2.05 per 1 Likert-scale point lower (95%CI 1.59–2.63) of having a H-TDB, and this association was confirmed also at multivariable analysis (aOR = 1.75, 95%CI 1.32–2.32). Additionally, Physical Health (per 1 pt lower, aOR = 1.52; 95%CI 1.19–1.94), Mental Health (per 1 pt lower, aOR = 1.52; 95%CI 1.23–1.88) and Sexual Health (per 1 pt lower, aOR = 1.44; 95%CI 1.17–1.77) were all associated with H-TDB, after adjusting for age, current antiretroviral therapy (ART), current reported HIV-RNA, treatment satisfaction and interaction with physician (Table 3). 

### 3.6. Focus on Patients’ Interest about Long Acting Agents

57% of PLHIV declared interest in Long-Acting Agents: they were younger (*p* < 0.001), with a higher education level (*p* = 0.02), with more recent HIV diagnosis (*p* < 0.001), afraid to disclose serostatus (*p* = 0.002) with a higher proportion of subjects without any comorbidity (*p* < 0.001) and not fully satisfied with the ongoing ART (*p* < 0.001). There were no differences in the overall ‘HIV Treatment and Diseases Burden’ and in the different domains between PLHIV interested and “not interested” in iLAA. While after analyzing the single items, subjects interested in Long-Acting Agents declare higher issues in their daily organization for taking ART (*p* = 0.026), were more bothered by having to rely on the ART medications (*p* < 0.001) and more frequently felt frustrated by their HIV status (*p* = 0.041) (Table 4). 

## 4. Discussion

This survey represents the first evidence of a systematic measurement of the burden of treatment and disease in patients with HIV in Italy. The aspects that have a negative impact on the burden of treatment and disease are younger age, low satisfaction with ongoing treatment, the need for more discussion with the treating physician and self-declared low health status. These findings confirm how treatment and disease burden is a multidimensional concept including both objective and subjective elements such as the number of medications, time to administration and monitoring treatment (objective) vs. negative feelings such as guilt, hopelessness and fear relating to treatment (subjective) [19].

Younger age was correlated with higher disease burden in the present survey. This could be possibly explained by the fact that it is a self-perceived data and young PLHIV, highly probably with more recent diagnosis, can be more susceptible to the disease burden.

Moreover, this result, on one hand, may be affected by the relatively low median age of the analyzed population (49 years), while on the other hand, suggests that targeted interventions to reduce the burden of disease and treatment should be directed with a special regard to young populations. The median age of the population that acquires HIV infection in recent years in Italy is relatively low [26] and the youth population represents a type of fragile patients with regard to the risk of non-adherence to antiretroviral therapy. Intervention to reduce treatment burden in this population could have a positive impact on treatment adherence with significant consequences from both individual (health) and population (not transmission of HIV) perspective [27].

Not perfect satisfaction with current treatment represents another aspect significantly associated with high disease and treatment burden in this survey. This finding does not seem to be completely explained by the type of treatment regimen in place. While the use of 3 ARV regimen was found to be associated with a two-fold higher risk of burden compared to the use of two-drug regimens whether the three-drug regimens were considered (both multidrug and single tablet with booster), this correlation was lost when considering the 3 ARV regimen only in single tablet regimen. This result suggests that treatment satisfaction goes beyond the classic variables such as number of tablets but is also not completely correlated with the number of drugs included in the regimen, as other factors such as potential side effects, manageability of the drug regimen in patient’s life as well as the fitting of the regimen into patient’s lifestyle may give a more detailed and precise definition of treatment satisfaction, as well the personal relationship between the patient and the therapy assumed.

A relevant aspect frequently found to be associated with high treatment and disease burden is the health practitioner–patient relationship [19]. Frequently, is has been demonstrated that failure of health-care provider to give significant information regarding treatment aspects was associated with higher treatment burden [5]. Scarce communication between patients and health-care providers was likely to result in the use of multiple medications (polypharmacy), which was associated with treatment burden [28]. Moreover, in the context of HIV infection, the patient–practitioner therapeutic relationship has been well-established over time with positive impact on patients’ beliefs about medicine-taking behavior and it could be essential for implementing the deprescribing strategy of antiretroviral therapy (ARV deprescribing) as deeply discussed by Guaraldi et al. [29].

A high treatment and disease burden has been associated with poor quality of life in our survey. This argument is strictly related to what has been defined by Lazarus et al. [30], the ‘fourth 90′ of the testing and treatment UNAIDS target: reaching optimal health-related quality of life in 90% of PLHIV. To the best of our knowledge, this is the first demonstration of a correlation of burden of HIV disease and quality of life, and it suggests that a systematic measurement of the different quality-of-life aspects should necessarily be included in the management of the HIV patient. This is also in terms of early detection of any imbalance of treatment burden that may affect adherence to treatment.

Can a measure of the Patient Experience with Treatment and Self-Management (PETS) be implemented in clinical practice in order to better understand the personalization of treatment from the patient perspective? The issue is surely worthy to be taken into consideration, even though in our opinion a more specific tool, taking into consideration different drivers in different populations, needs to be developed.

Of note, not in the HIV field, while our survey was ongoing (March 2021), Mohammed A et al. [31] found an independent association between a Drug Burden Index and a poorer Psychological wellbeing and Functional and Role Limitation domains of the Medication-Related Burden Quality of Life (MRB-QoL), even though they state that since the types and severity of medical conditions and the clinical appropriateness of medications were not evaluated, these factors can have affected the associations. The results of the present survey suggest how the measurement of treatment and disease burden could be useful to understand several health care performances.

It could provide a novel, patient-centered outcome measure for comparative effectiveness studies [32,33], which are still lacking in HIV infection such as in many other chronic conditions.

It could identify specific subgroup of patients with poor health outcomes for whom a tailored, patient-centered approach could be appropriate also in a cost-effective way, such as medical homes, nurse-coordinated care, remote care management [33].

Moreover, health providers could receive information to identify patients who may have low adherence to treatment or to care system and may benefit from a minimally invasive ARV drug regimen [29].

### Limitations

The present study has some limitations. First of all, we cannot rule out that some associations found are due to collider bias: age, education level and interest in health awareness are indeed all factors influencing the inclusion in voluntary samples selected through online surveys. Secondly, clinical data on HIV and ARV therapy are only self-reported by the survey participants and are not checked with medical records of the subjects, given the anonymous collection. Further details on expectation, perceived benefits and limits of iLAA or other future perspectives of HIV therapy has not been investigated in this survey.

## 5. Conclusions

Developing measurements that comprehensively assesses the domains of treatment burden is still lacking in HIV infection and should be included in the research agenda in order to develop a more tailored patient-centered approach linked to the improvement of health-related quality of life.

## Figures and Tables

**Table 1:** Sociodemographic and clinical characteristics of PLHIV according with level of disease and treatment burden.

Characteristics	L-TBD (*n* = 432; 81.3%)	H-TDB (*n* = 99; 18.6%)	*p*-Value	Total
**Age, years, median (IQR)**	50 (41–57)	44 (34–53)	<0.001	49 (39–56)
Sex *, Male, ***n*** **(%)**	375 (86.8)	87 (87.9)	0.775	462 (87.0)
**Education, ** ***n*** **(%)**			0.109	
Middle/High School	254 (58.8)	50 (50.5)		304 (57.2)
University/Master	176 (40.7)	47 (47.5)		223 (42.0)
Other/Unknown	2 (0.5)	2 (2.0)		4 (0.7)
**Job, ** ***n*** **(%)**			0.102	
Stable work	272 (62.9)	52 (52.5)		324 (60.0)
Not stable work	55 (12.7)	15 (15.1)		70 (13.2)
Not working	44 (10.2)	18 (18.2)		62 (11.7)
Other/Unknown/Retired	61 (14.1)	14 (14.1)		75 (14.1)
**Italy Born, ** ***n*** **(%)**	406 (94.0)	92 (92.9)	0.696	498 (93.8)
**Year HIV Diagnosis, ** ***n*** **(%)**			0.178	
<2001	106 (24.5)	23 (23.2)		129 (24.3)
2001–2010	114 (26.4)	18 (18.2)		132 (24.9)
2011–2015	108 (25)	25 (25.2)		133 (25)
>2015	104 (24.1)	33 (33.3)		137 (25.8)
**Year first ARV start, ** ***n*** **(%)**			0.137	
<2001	89 (20.6)	17 (17.17)		106 (19.9)
2001–2010	109 (25.23)	17 (17.17)		126 (23.7)
2011–2015	116 (26.85)	28 (28.28)		144 (27.1)
>2015	118 (27.31)	37 (37.37)		155 (29.2)
**Mode of HIV** **transmission, ** ***n*** **(%)**			0.194	
Homosexual Contacts	274 (63.4)	52 (52.5)		326 (61.4)
Heterosexual Contacts	31 (7.2)	7 (7.1)		38 (7.2)
Sexual contacts (mode not specified)	88 (20.4)	27 (27.3)		115 (21.7)
Other not-sexual mode	39 (9.0)	13 (13.1)		52 (9.8)
**Current HIV-RNA,** ** copies/mL, ** ***n*** **(%)**			0.044	
<50 copiess/mL	388 (89.8)	80 (80.8)		468 (88.1)
≥50 copies/mL	19 (4.4)	8 (8.1)		27 (5.1)
unknown/not reported	25 (5.8)	11 (11.1)		36 (6.8)
**Current CD4, cells/mmc, ** ***n*** **(%)**			0.463	
<200 cells/mmc	39 (9.0)	12 (12.12)		51 (9.6)
200–500 cells/mmc	87 (20.14)	18 (18.18)		105 (19.8)
>500 cells/mmc	251 (58.1)	52 (52.53)		303 (57.1)
unknown/not reported	55 (12.73)	17 (17.17)		72 (13.6)
**Current Regimen (R), ** ***n*** **(%)**			0.011	
2 ARV R STR	85 (19.68)	13 (13.13)		98 (18.46)
3 ARV R STR (w/o booster)	203 (46.99)	46 (46.46)		249 (46.9)
3 ARV R STR (with booster)	32 (7.41)	14 (14.14)		46 (8.66)
2 ARV R MTR	58 (13.43)	8 (8.08)		66 (12.4)
3 ARV R MTR	37 (8.56)	7 (7.07)		44 (8.3)
Unknown	17 (3.9)	11 (11.1)		28 (5.3)
**STR, ** ***n*** **(%)**	320 (77.1)	73 (82.9)	0.228	393 (78.1)
**Treatment Satisfaction, ** ***n*** **(%)**			0.007	
Yes, fully	243 (56.25)	26 (26.26)		269 (50.66)
Yes, but can be improved	180 (41.67)	61 (61.62)		241 (45.39)
No, I’ve some issues	9 (2.08)	7 (7.07)		16 (3.01)
Not at all	0 (0.0)	2 (2.02)		2 (0.38)
Other/Unknown	0 (0.0)	3 (3.0)		3 (0.6)
**Number of daily assumption of all the drugs > 1, ** ***n*** **(%)**	106 (24.5)	26 (26.3)	0.72	132 (24.9)
**Polypharmacy, ≥5 drugs/day, ** ***n*** **(%)**	75 (17.4)	18 (18.2)	0.846	93 (17.5)
**Physical Health, (1–5 scale), median (IQR)**	2 (1–3)	3 (2–3)	<0.001	2 (1–3)
**Mental Health, (1–5 scale), median (IQR)**	2 (1–3)	3 (2–4)	<0.001	2 (2–3)
**Sexual Health, (1–5 scale), median (IQR)**	3 (2–4)	4 (3–5)	<0.001	3 (3–5)
**Overall Health, (1–5 scale), median (IQR)**	2 (2–3)	3 (2–3)	<0.001	2 (2–3)

Abbreviations: 2 ARV R (2-drug antiretroviral regimen), 3 ARV R (3-drug antiretroviral regimen), STR (single tablet regimen), MTR (multiple tablet regimen), w/o (without). * Sex at birth.

**Table 2:** Factors associated with high HIV treatment and disease burden (H-TDB).

	OR	95%CI		*p*	aOR *	95%CI		*p*
**Age (per 10 years younger)**	1.46	1.19	1. 79	<0.001	1.43	1.14	1.80	0.002
**Current HIV-RNA**								
<50 cps/mL	1.00				1.00			
≥50 cps/mL	2.04	0.86	4.83	0.104	1.96	0.74	5.20	0.174
unknown	2.13	1.01	4.51	0.047	1.29	0.49	3.37	0.606
**Current Regimen (R)**								
2 ARV R STR or MTR	1.00				1.00			
3 ARV R STR (w/o booster)	1.54	0.88	2.70	0.128	1.57	0.75	3.31	0.227
3 ARV R MTR or 3 ARV R STR w booster	2.07	1.06	4.05	0.033	1.44	0.79	2.62	0.234
Unknwon	4.41	1.82	10.69	0.001	3.20	1.11	9.24	0.031
**Treatment Satisfaction**								
Yes, fully	1.00				1.00			
Yes, but can be improved	3.17	1.93	5.21	<0.001	2.39	1.40	4.09	0.001
No, I’ve some issues	7.27	2.50	21.14	<0.001	3.51	1.04	11.80	0.043
**I would like to have a more sincere dialogue with my Infectious Diseases physician (vs. No)**	3.97	2.30	6.87	<0.001	2.75	1.49	5.05	0.001
**>1 daily assumption of all the drugs**	1.10	0.67	1.80	0.720				
**Polypharmacy,** ≥ **5 drugs/die**	1.06	0.60	1.87	0.846				
**Other comorbidities**	1.14	0.71	1.83	0.585				
**Overall Health, per 1 point lower (1–5 scale)**	2.05	1.59	2.63	<0.001	1.75	1.32	2.32	<0.001

* Adjusted for overall Health status, dialogue with Infectious Diseases (ID) physician, treatment satisfaction, current reported ART, current reported HIV-RNA and age.

**Table 3:** Association with Health Status and high HIV treatment and disease burden (H-TDB).

	OR	95%CI		*p*	AOR *	95%CI		*p*
**Overall Health Status, per 1-pt lower**	2.05	1.59	2.63	<0.001	1.76	1.32	2.33	<0.001
**Physical Health Status, per 1-pt lower**	1.76	1.42	2.19	<0.001	1.52	1.19	1.95	0.001
**Mental Health Status, per 1-pt lower**	1.74	1.43	2.11	<0.001	1.53	1.23	1.89	<0.001
**Sexual Health Status, per 1-pt lower**	1.55	1.29	1.86	<0.001	1.44	1.18	1.77	<0.001

* Four different models fitted with the following covariates: dialogue with ID, treatment satisfaction, current reported ART, current reported HIV-RNA and age.

**Table 4:** Single items, domains and total results of “HIV treatment and diseases burden” questionnaire adapted from DT Eton et al., Qual Life Res 2017, according to interest in injecting long acting (mean and t-test for items, weighted mean and weighted linear regression for domains and total).

	No Interest in iLLA			Interest in iLLA			
	N	(Weighted) Mean	95%CI	N	(Weighted) Mean	95%CI	*p*
**Medical Information**		**2.31**	**[2.20–2.42]**		**2.37**	**[2.17–2.57]**	**0.625**
Find info on HIV status	224	2.21	[2.06–2.35]	304	2.17	[2.06–2.28]	0.711
Find info on HIV treatment	222	2.33	[2.19–2.46]	294	2.33	[2.21–2.45]	0.990
Understand changes on HIV treatment	213	2.32	[2.18–2.47]	285	2.32	[2.20–2.44]	0.991
Understand reasons for taking certain HIV treatment	219	2.42	[2.27–2.58]	294	2.56	[2.43–2.69]	0.179
Find reliable sources on HIV and HIV treatment	222	2.45	[2.31–2.59]	291	2.70	[2.56–2.83]	0.015
Understand physician’s suggestion	224	2.14	[2.01–2.27]	302	2.12	[2.01–2.23]	0.823
**HIV Medication**		**1.84**	**[1.45–2.23]**		**2.07**	**[1.77–2.37]**	**0.311**
Daily organization	219	1.74	[1.59–1.89]	292	1.98	[1.83–2.12]	0.026
Taking HIV medication once a day	218	1.44	[1.32–1.57]	291	1.76	[1.63–1.89]	0.001
Taking HIV medication more times a day	117	1.95	[1.70–2.19]	147	2.18	[1.94–2.43]	0.188
Supplying of HIV drugs	218	2.22	[2.04–2.40]	291	2.34	[2.17–2.52]	0.345
**Bother to HIV medication**		**2.43**	**[2.35–2.51]**		**2.83**	**[2.16–3.50]**	**0.227**
Dependence on HIV drugs	214	2.41	[2.22–2.59]	288	3.04	[2.86–3.21]	<0.001
Side effects of HIV drugs	206	2.46	[2.26–2.65]	279	2.62	[2.43–2.80]	0.247
**Appointments/practices HIV**		**1.99**	**[1.86–2.12]**		**2.05**	**[1.94–2.15]**	**0.420**
Bureaucracy	198	1.92	[1.74–2.10]	255	2.08	[1.91–2.25]	0.213
Booking appointments	209	2.15	[1.96–2.33]	280	2.16	[2.00–2.33]	0.922
Circularity of health info among HCPs	200	1.95	[1.76–2.13]	268	1.96	[1.81–2.12]	0.884
Waiting time for drugs supply	206	1.95	[1.77–2.12]	277	2.00	[1.83–2.16]	0.685
**Interpersonal Challenges**		**2.07**	**[1.66–2.49]**		**2.28**	**[1.82–2.74]**	**0.465**
Dependence for HIV management from others	189	2.02	[1.83–2.22]	256	2.27	[2.09–2.45]	0.069
Other people who remind you things on HIV health	177	1.62	[1.46–1.77]	241	1.75	[1.59–1.91]	0.244
Stress in relationship with others	188	2.21	[2.00–2.42]	252	2.42	[2.24–2.61]	0.129
Misunderstanding with others	187	2.44	[2.22–2.67]	248	2.67	[2.47–2.87]	0.134
**Limitations of role and social activity**		**1.68**	**[1.58–1.77]**		**1.81**	**[1.68–1.94]**	**0.116**
Work	188	1.62	[1.46–1.78]	268	1.75	[1.60–1.90]	0.236
Family responsabilities	193	1.74	[1.56–1.91]	269	1.94	[1.78–2.10]	0.099
Daily activities	202	1.63	[1.49–1.78]	278	1.84	[1.70–1.99]	0.051
Freetime activity	201	1.66	[1.51–1.81]	279	1.75	[1.61–1.88]	0.401
Spending time with family	202	1.55	[1.40–1.70]	278	1.59	[1.46–1.71]	0.708
Travelling	196	1.86	[1.68–2.05]	274	1.99	[1.83–2.16]	0.305
**Physical and mental exhaustion**		**2.42**	**[2.13–2.71]**		**2.58**	**[2.28–2.88]**	**0.417**
Angry	201	2.42	[2.25–2.60]	278	2.63	[2.48–2.77]	0.075
Worried	202	2.81	[2.66–2.96]	278	2.97	[2.83–3.11]	0.128
Depressed	200	2.53	[2.36–2.70]	277	2.60	[2.46–2.74]	0.542
Exhausted	199	2.03	[1.86–2.19]	277	2.14	[2.00–2.29]	0.279
Frustrated	199	2.33	[2.15–2.50]	277	2.57	[2.42–2.72]	0.041
**Total HIV therapy burden score**		**2.09**	**[2.08–2.10]**		**2.24**	**[2.23–2.24]**	**0.109**

Data reported in bold refers to the weighted median, 95%CI and p-values of the 7 different domains.

## Data Availability

The data presented in this study are available on request from the corresponding author.

## Supplementary Materials

The following are available online at https://www.mdpi.com/article/10.3390/jcm11020471/s1, Understanding the experience of people with HIV about treatment and health management from their own experience (survey for people living with HIV on antiretroviral therapy). Membership of the Icona Foundation Study Group.
