# Supplementary material for: Correlates of Treatment and Disease Burden in People Living with HIV (PLHIV) in Italy
Source: J Clin Med. 2022 Jan 17;11(2):471. doi: 10.3390/jcm11020471 (PMC8781185; doi:10.3390/jcm11020471)
Supplement: Supplementary file 1 [file jcm-11-00471-s001.zip › jcm-11-00471-s001/jcm-1474181 supplementary 2.pdf]

The Authors would like to thank the participants to the survey, the Italian Patient Advocacy Groups and the ICONA Foundation Study Group as listed below. Italian Patient Advocacy Groups: ANLAIDS Onlus (https://www.anlaidsonlus.it); ASA Onlus (http://www.asamilano30.org); Circolo di Cultura Omosessuale Mario Mieli (https://www.mariomieli.net); Associazione Arcobaleno AIDS ODV (https://www.arcobalenoaids.it); NPS Italia Onlus (https://www.npsitalia.net); CNCA (https://www.cnca.it); Associazione Nadir Onlus (https://www.nadironlus.org); Fondazione The Bridge (https://www.fondazionethebridge.it); PLUS—Rete persone LGBT+ sieropositive APS (https://www.plus-aps.it); LILA Onlus (https://www.lila.it/). ICONA Foundation Study Group: BOARD OF DIRECTORS: A d'Arminio Monforte (President), A Antinori (Vice-President), M Andreoni, A Castagna, F Castelli, R Cauda, G Di Perri, M Galli, R Iardino, G Ippolito, A Lazzarin, GC Marchetti, G Rezza, F von Schloesser, P Viale. SCIENTIFIC SECRETARY: A d'Arminio Monforte, A Antinori, A Castagna, F Ceccherini-Silberstein, A Cozzi-Lepri, E Girardi, A Gori, S Lo Caputo, F Maggiolo, C Mussini, M Puoti, CF Perno. STEERING COMMITTEE: A Antinori, F Bai, A Bandera, S Bonora, M Borderi, A Calcagno, MR Capobianchi, A Castagna, F Ceccherini-Silberstein, S Cicalini, A Cingolani, P Cinque, A Cozzi-Lepri, A d'Arminio Monforte, A Di Biagio, R Gagliardini, E Girardi, N Gianotti, A Gori, G Guaraldi, G Lapadula, M Lichtner, A Lai, S Lo Caputo, G Madeddu, F Maggiolo, G Marchetti, C Mussini, S Nozza, CF Perno, S Piconi, C Pinnetti, M Puoti, E Quiros Roldan, R Rossotti, S Rusconi, MM Santoro, A Saracino, L Sarmati, V Spagnuolo, V Svicher, L Taramasso. STATISTICAL AND MONITORING TEAM: A Cozzi-Lepri, I Fanti, A Rodano', A Tavelli. COMMUNITY ADVISORY BOARD: A Bove, A Camposeragna, M Errico, M Manfredini, A Perziano, V Calvino. BIOLOGICAL BANK INMI: F Carletti, S Carrara, A Di Caro, S Graziano, F Petroni, G Prota, S Truffa. PARTICIPATING PHYSICIANS AND CENTERS: Italy A Giacometti, A Costantini, V Barocci (Ancona); A Saracino, L Monno, E Milano (Bari); F Maggiolo, C Suardi (Bergamo); P Viale, V Donati, G Verucchi (Bologna); E Quiros Roldan, C Minardi, (Brescia); B Menzaghi, C Abeli (Busto Arsizio); L Chessa, F Pes (Cagliari); P Maggi, L Alessio (Caserta); B Cacopardo, B Celesia (Catania); J Vecchiet, K Falasca (Chieti); A Pan, S Dal Zoppo (Cremona); L Sighinolfi, D Segala (Ferrara); F Vichi, MA Di Pietro (Firenze); T Santantonio, S Ferrara (Foggia); M Bassetti, E Pontali, A Alessandrini, N Bobbio, G Mazzarello (Genova); M Lichtner, L Fondaco (Latina); S Piconi, C Molteni (Lecco); A Chiodera, P Milini (Macerata); G Nunnari, G Pellicanò (Messina); A d'Arminio Monforte, M Galli, A Lazzarin, G Rizzardini, M Puoti, A Gori, A Castagna, V Bono, MC Moioli, R Piolini, D Bernacchia, A Poli, C Tincati (Milano); C Mussini, C Puzzolante (Modena); P Bonfanti, G Lapadula (Monza); V Sangiovanni, I Gentile, V Esposito, G Di Flumeri, G Di Filippo, V Rizzo (Napoli); AM Cattelan, S Marinello (Padova); A Cascio, C Colomba (Palermo); D Francisci, E Schiaroli (Perugia); G Parruti, F Sozio (Pescara); P Blanc, A Vivarelli (Pistoia); C Lazzaretti, R Corsini (Reggio Emilia); M Andreoni, A Antinori, R Cauda, C Mastroianni, V Mazzotta, S Lamonica, M Capozzi, A Mondi, A Cingolani, M Rivano Capparuccia, G Iaiani, C Stingone, L Gianserra, G Onnelli, MM Plazzi, G d'Ettore, A Vergori (Roma); M Cecchetto, F Viviani (Rovigo); G Madeddu, A De Vito (Sassari); M Fabbiani, F Montagnani (Siena); A Franco, R Fontana Del Vecchio (Siracusa); BM Pasticci, C Di Giuli (Terni); GC Orofino, G Calleri, G Di Perri, S Bonora, M Sciandra (Torino); C Tascini, A Londero (Udine); V Manfrin, G Battagin (Vicenza); G Starnini, A Ialungo (Viterbo).
