# Supplementary material for: Correlates of Treatment and Disease Burden in People Living with HIV (PLHIV) in Italy
Source: J Clin Med. 2022 Jan 17;11(2):471. doi: 10.3390/jcm11020471 (PMC8781185; doi:10.3390/jcm11020471)
Supplement: Supplementary file 1 [file jcm-11-00471-s001.zip › jcm-11-00471-s001/jcm-1474181-supplementary.pdf]

Italian version of the Survey

CAPIRE DAL VISSUTO DELLA PERSONA CON HIV L'ESPERIENZA SULLA TERAPIA E SULLA GESTIONE DELLA SALUTE

INDAGINE RIVOLTA A PERSONE CON HIV CHE ASSUMONO TERAPIA ANTIRETROVIRALE

1. Caratteristiche sociodemografiche

CHI SEI?
PUOI DIRCI QUALCHE COSA DI TE

Anno di nascita: …………

Sesso registrato alla nascita all'anagrafe

Maschio	☐
Femmina	☐

Come percepisci la tua identità di genere?

Uomo	☐
Donna	☐
Altra identità	☐

Come ti identifichi?

Cisgender*<br>*persona che si identifica con il sesso assegnato alla nascita	☐
Transgender	☐

Come descriveresti il tuo orientamento sessuale?

Omosessuale	☐
Bisessuale	☐
Eterosessuale	☐
Non specificato	☐

Quale livello di educazione hai completato?

Scuola Media inferiore	☐
Scuola Media Superiore	☐
Università	☐
Master post-Laurea	☐
Altro/non so/non voglio rispondere	☐

Nazione di nascita: ………….

A quale gruppo etnico appartieni?

Bianco/caucasico	☐
Afroamericano/africano	☐
Asiatico	☐
Latino-americano	☐
Etnia mista	☐
Altro/non so/non voglio rispondere	☐

Regione di residenza

Abruzzo	☐	Molise	☐
Basilicata	☐	Piemonte	☐

Calabria	☐	Puglia	☐
Campania	☐	Sardegna	☐
Emilia-Romagna	☐	Sicilia	☐
Friuli-Venezia Giulia	☐	Toscana	☐
Lazio	☐	Trentino-Alto Adige	☐
Liguria	☐	Umbria	☐
Lombardia	☐	Valle d'Aosta	☐
Marche	☐	Veneto	☐

Quale situazione descrive al meglio, ora, il tuo *status relazionale*?

Single (solo)	☐
Sposato/a o unito/a civilmente	☐
“Ho una relazione e ci convivo”	☐
“Ho una relazione, ma non convivo”	☐
Vedovo/a	☐
Divorziato/a	☐
Altro/non so/non voglio rispondere	☐

Quale delle seguenti affermazioni descrive al meglio, ora, il tuo *status lavorativo*?

Lavoro stabilmente	☐
Lavoro, ma sono (o mi sento) precario	☐
Non lavoro, perché non lo trovo, ma lo cerco	☐
Non lavoro e non lo cerco	☐
Pensionato/a	☐
Altro/non so/non voglio rispondere	☐

2. Caratteristiche sull’HIV della persona

TI CHIEDIAMO ORA QUALCHE DATO SUL TUO HIV E LA TUA SALUTE

QUANDO HAI AVUTO LA DIAGNOSI DI “HIV-POSITIVITÀ”?

Prima del 2000	☐
Dal 2001 al 2010	☐
Dal 2011 al 2015	☐
Dal 2016 in poi	☐

COME PENSI DI AVERE ACQUISITO L’INFEZIONE DA HIV?

Rapporti sessuali con persone del mio stesso sesso (da intendersi “alla nascita”)	☐
Rapporti sessuali con persone di sesso diverso (da intendersi “alla nascita”)	☐
Rapporti sessuali (non specificato)	☐
Altre modalità, <u>non sessuali</u>	☐

QUANDO HAI INIZIATO LA TERAPIA ANTI-HIV?

Prima del 2000	☐
Dal 2001 al 2010	☐
Dal 2011 al 2015	☐
Dal 2016 in poi	☐

RICORDI IL TUO ULTIMO VALORE DI CELLULE CD4 (per ml)?

MENO DI 200	☐
DA 200 A 500	☐
PIU' DI 500	☐
NON RICORDO	☐

RICORDI IL TUO ULTIMO VALORE DI CARICA VIRALE (OSSIA HIV-RNA)?

NON RILEVATO (undetectable)	☐
RILEVATO TRA 50 E 200 (cp/mL)	☐
RILEVATO OLTRE I 200 (cp/mL)	☐
NON RICORDO	☐

QUALI FARMACI ASSUMI PER L'HIV?
Nota alla lettura: una compressa, ossia un 'farmaco' con un determinato nome commerciale, può contenere uno o più 'principi attivi'.

DUE PRINCIPI ATTIVI IN UNA COMPRESSA (Dovato®, Juluca®)	☐
TRE PRINCIPI ATTIVI IN UNA COMPRESSA (Atripla®, Biktarvy®, Delstrigo®, Eviplera®, Odefsey®, Triumeq®)	☐
QUATTRO PRINCIPI IN UNA COMPRESSA (Genvoya®, Stribild®, Symtuza®)	☐
DUE PRINCIPI ATTIVI IN DUE COMPRESSE	☐
TRE/QUATTRO PRINCIPI IN DUE/TRE COMPRESSE – TUTTI FARMACI IN COMMERCIO	☐
ASSUMO ANCHE FARMACI ANTI-HIV CHE NON SONO ANCORA IN COMMERCIO (SPERIMENTALI)	☐
ALTRO	☐
NON RICORDO	☐

SEI SODDISFATTO DELLA TUA TERAPIA ANTI-HIV?

Sì, completamente	☐
Sì, ma penso si possa migliorare	☐
No, perché ho problemi	☐
No, non lo sono per niente	☐
Altro/non so/non voglio rispondere	☐

PERCHÉ HAI CAMBIATO LA TERAPIA L'ULTIMA VOLTA?

Quella che prendo è la mia prima terapia, non l'ho mai cambiata	☐
Perché non stavo bene, non la tolleravo (effetti collaterali) e ne ho parlato col medico	☐
Perché gli esami biochimici (cioè il controllo del fegato, dei reni, il colesterolo, i trigliceridi, ecc..) non andavano bene e il medico me l'ha cambiata	☐
Perché il mio medico mi ha proposto un'altra terapia senza particolari ragioni	☐
Perché prendevo troppe compresse o perché la prendevo più volte al giorno	☐
Perché da tre principi attivi (anche in una sola compressa) sono passato a due principi attivi (anche in una sola compressa)	☐
Perché da due principi attivi (anche in una sola compressa) sono passato a tre principi attivi (anche in una sola compressa)	☐
Perché la carica virale era cresciuta e quindi c'era un problema di efficacia	☐
Altro/non so/non voglio rispondere	☐

DELL'HIV PARLO CON…*(possibili più risposte)*

Tutti	☐
I miei colleghi	☐
La maggior parte di amici/amiche	☐

La mia famiglia	☐
Il mio/la mia partner	☐
Qualche (anche solo uno/a) amico/a particolare	☐
Il mio medico/infermiere/a e nessun altro/a	☐
Altro/non so/non voglio rispondere	☐

VORREI…*(possibili più risposte)*

Avere più voce in capitolo nello scegliere la mia terapia anti-HIV	☐
Poter assumere la terapia anti-HIV non tutti i giorni, grazie a farmaci appositamente studiati	☐
Avere un dialogo più frequente con il mio medico infettivologo	☐
Essere più controllato su alcuni aspetti medici	☐
Parlare di più con qualcuno (un professionista) non solo degli aspetti medici dell'HIV	☐
Conoscere quando arriveranno dei nuovi farmaci e perché	☐
Avere un dialogo più sincero con il mio medico infettivologo	☐
Nulla di quanto sopra	☐

3. Caratteristiche sulla salute in generale

TI CHIEDIAMO ORA QUALCHE DATO SU ALTRI ASPETTI DELLA TUA SALUTE…

NELL'ULTIMO MESE, COME DESCRIVERESTI IL TUO STATO DI SALUTE?

	MOLTO BUONO	ABBASTANZA BUONO	NORMALE	SCARSO	MOLTO INSODDIFACENTE
Salute fisica	☐	☐	☐	☐	☐
Salute mentale	☐	☐	☐	☐	☐
Salute sessuale	☐	☐	☐	☐	☐
Salute 'complessiva'	☐	☐	☐	☐	☐

CONSIDERANDO TUTTI I FARMACI O CIO' CHE <u>PRENDI IN MODO CONTINUATIVO</u> (sia anti-HIV sia per eventuali altre co-patologie sia per altro), QUANTE PILLOLE, BUSTINE, ECC…PRENDI OGNI GIORNO?

☐ 1 ☐ 2 ☐ 3 ☐ 4 ☐ 5 ☐ 6 ☐ 7 ☐ 8 ☐ 9 ☐ 10 ☐ Più di 10

… E QUANTE VOLTE AL GIORNO?

☐ 1 ☐ 2 ☐ 3 ☐ 4 ☐ 5 ☐ Più di 5

PER TE….

…l'HIV è il problema di salute (cioè la patologia) più importante	☐
…l'HIV non è il problema di salute (cioè la patologia) più importante	☐
Non so/non voglio rispondere	☐

FACCIO ATTIVITÀ FISICA/SPORT ….

In modo continuativo (2 volte alla settimana)	☐
2-4 volte al mese	☐
No, non la faccio	☐
Altro/non so/non voglio rispondere	☐

MANGIO….

In modo controllato, cibi sani, seguendo un certo regime alimentare almeno 5 giorni su 7	☐
In modo controllato, cibi sani, seguendo un certo regime alimentare almeno 2 giorni su 7	☐
In modo non controllato	☐
Altro/non so/non voglio rispondere	☐

BEVO ALCOLICI….

In modo controllato, in compagnia, un paio di bicchieri 2 giorni su 7	☐
Un po' come capita, in compagnia, un paio di bicchieri 4/5 giorni su 7	☐
In modo non controllato	☐
Altro/non so/non voglio rispondere	☐

4. Carico del trattamento dell'HIV

RACCONTA LA TUA ESPERIENZA CON LA TERAPIA ANTI-HIV, CON L'ASSISTENZA E CON L'HIV

Informazioni mediche riguardo l'HIV e i farmaci anti-HIV

In merito "ai tempi di riferimento" di queste domande, riferisciti, se pertinente, a una media delle tue esperienze **negli ultimi 6 mesi.**

QUANTO PER TE È STATO FACILE/DIFFICILE….

	Molto Facile	Facile	Né facile né difficile	Difficile	Molto Difficile	Non mi riguarda
Acquisire informazioni sul tuo stato di salute dal medico infettivologo che ti segue per l'HIV						
Trovare le informazioni che desideravi conoscere riguardo ai farmaci anti-HIV che assumi						
Comprendere gli eventuali cambiamenti del tuo piano terapeutico fornito dall'infettivologo che ti segue per l'HIV						
Capire le ragioni per le quali assumi certi farmaci anti-HIV e non altri						
Trovare fonti per te affidabili per avere informazioni mediche sia su vari aspetti dell'HIV sia sui farmaci anti-HIV						
Capire i suggerimenti e/o le indicazioni dal medico infettivologo che ti segue per l'HIV						

Farmaci anti-HIV

In merito "ai tempi di riferimento" di queste domande, riferisciti, se pertinente, a una media delle tue esperienze **nell'ultimo mese**.

QUANTO PER TE È STATO UN PROBLEMA….

	Non lo è stato	Lo è stato poco	Né poco né molto	Lo è stato un po'	Lo è stato molto	Non mi riguarda
Organizzarsi praticamente, nel quotidiano, con i farmaci anti-HIV (conservazione, assunzione, …)						
Assumere i farmaci anti-HIV una volta al giorno						
Assumere i farmaci anti-HIV più volte al giorno						
Rifornirsi dei farmaci anti-HIV in						

farmacia ospedaliera (o sul territorio se così previsto nella regione di appartenenza)						

Indicatori "singoli" di fastidio ai farmaci anti-HIV
In merito "ai tempi di riferimento" di queste domande, riferisciti, se pertinente, a una media delle tue esperienze **nell'ultimo mese**.

QUANTO SEI STATO/A INFASTIDITO/A…

	**Non lo sono stato**	**Lo sono stato poco**	**Né poco né molto**	**Lo sono stato un po'**	**Lo sono stato molto**	**Non mi riguarda**
…da quanto devi dipendere dai tuoi farmaci?						
…dagli effetti collaterali dei tuoi farmaci?						

Appuntamenti/pratiche riguardanti gli aspetti medici per l'HIV
In merito "ai tempi di riferimento" di queste domande, riferisciti, se pertinente, a una media delle tue esperienze **negli ultimi 6 mesi**.

QUANTO PER TE È STATO UN PROBLEMA….

	**Non lo è stato**	**Lo è stato poco**	**Né poco né molto**	**Lo è stato un po'**	**Lo è stato molto**	**Non mi riguarda**
…compilare moduli (cioè 'burocrazia') per l'assistenza sanitaria riguardo l'HIV						
…prenotare i tuoi appuntamenti con il medico infettivologo che ti segue per l'HIV in orari a te convenienti						
…assicurarti che quanto deciso con il medico infettivologo fosse poi recepito da altri operatori sanitari (es.: infermieri, farmacisti, altri medici specialisti)						
…aspettare troppo a lungo alla farmacia ospedaliera (o sul territorio se così previsto nella regione di appartenenza) per ritirare i farmaci anti-HIV						

Sfide interpersonali (legate all'HIV)
In merito "ai tempi di riferimento" di queste domande, riferisciti, se pertinente, a una media delle tue esperienze **nell'ultimo mese**.

QUANTO SEI STATO INFASTIDITO/A DA…

	Non lo sono stato	Lo sono stato poco	Né poco né molto	Lo sono stato un po’	Lo sono stato molto	Non mi riguarda
…sentirsi dipendenti da altre persone per le proprie esigenze sanitarie legate all’HIV						
…altre persone che ti ricordano di fare cose per la tua salute in merito all’HIV come prendere medicine, programmare appuntamenti medici...						
…i tuoi bisogni sanitari riguardo all’HIV, perché creano tensione nel rapporto con le altre persone						
…altre persone, perché non capiscono la tua situazione riguardo all’HIV						

Limitazioni del ruolo e dell’attività sociale (in ambito HIV)
In merito “ai tempi di riferimento” di queste domande, riferisciti, se pertinente, a una media delle tue esperienze **nell’ultimo mese**.

QUANTO L’HIV, NELLA CURA DI TE STESSO/A, HA INTERFERITO CON…

	Non ha interferito	Un po’…	Né sì né no	Abbastanza	Ha interferito molto	Non mi riguarda
Il lavoro (compreso il lavoro da casa)						
Le responsabilità famigliari						
Le attività quotidiane						
Le attività del tempo libero						
La capacità di trascorrere del tempo con la famiglia e/o amici						
La capacità di viaggiare per lavoro, vacanza, …						

Esaurimento fisico e mentale (in ambito HIV)
In merito “ai tempi di riferimento” di queste domande, riferisciti, se pertinente, a una media delle tue esperienze **nell’ultimo mese**.

QUANTE VOLTE L’HIV, NELLA CURA DI TE STESSO/A, TI HA FATTO SENTIRE…

	Mai	Raramente	Qualche volta	Spesso	Sempre	Non mi riguarda
Arrabbiato/a						
Preoccupato/a						
Depresso/a						
Sfinito/a						
Frustrato/a						

English translation of the survey

UNDERSTANDING THE EXPERIENCE OF PEOPLE WITH HIV ABOUT TREATMENT AND HEALTH MANAGEMENT FROM THEIR OWN EXPERIENCE

SURVEY FOR PEOPLE LIVING WITH HIV ON ANTIRETROVIRALTHERAPY

1. Socio-demographic characteristics

WHO ARE YOU?
CAN YOU TELL US SOMETHING ABOUT YOURSELF

Year of birth: …………

Gender at birth

Male	☐
Female	☐

How do you perceive your gender identity?

Male	☐
Female	☐
Other	☐

How you identify yourself?

Cisgender*<br>**person who identifies with sex assigned at birth*	☐
Transgender	☐

How would you describe your sexual orientation?

Homosexual	☐
Bisexual	☐
Eterosexual	☐
Not specified	☐

What is the highest level of education completed?

Lower secondary school	☐
Higher secondary school	☐
Degree	☐
Post-graduateter ‘s degree	☐
Other/don’t know/don’t want answer	☐

Country of birth: ………….

What ehtnic group do you belong to?

Caucasian	☐
Afro-american	☐
Asiatic	☐
Latin-american	☐
Mixed	☐
Other/don’t know/don’t want answer	☐

Region of residency

Abruzzo	☐	Molise	☐
Basilicata	☐	Piemonte	☐
Calabria	☐	Puglia	☐
Campania	☐	Sardegna	☐

Emilia-Romagna	☐	Sicilia	☐
Friuli-Venezia Giulia	☐	Toscana	☐
Lazio	☐	Trentino-Alto Adige	☐
Liguria	☐	Umbria	☐
Lombardia	☐	Valle d'Aosta	☐
Marche	☐	Veneto	☐

Which situation best describes your relational status now?

Single (only)	☐
Married or civilly united	☐
"I'm in a relationship and living with someone	☐
"I'm in a relationship, but I'm not living with someone".	☐
Widowed	☐
Divorced	☐
Other / don't know / don't want to answer	☐

Which of the following statements best describes your job status now?

I work permanently	☐
I work, but I am (or feel) precarious	☐
I don't work because I can't find it, but I'm looking for it	☐
Not working and not looking for work	☐
Retired	☐
Other / don't know / don't want to answer	☐

2. HIV characteristics

NOW WE ASK YOU SOME DATA ABOUT YOUR HIV AND YOUR HEALTH

When did you receive your diagnosis of HIV infection?

Before 2000	☐
2001 to 2010	☐
2011 to 2015	☐
2016 onwards	☐

How do you think you acquired the HIV infection?

Sexual intercourse with persons of the same sex (to be understood as 'at birth')	☐
Sexual intercourse with persons of a different sex (to be understood as 'at birth')	☐
Sexual intercourse (not specified)	☐
Other modes, non-sexual	☐

When did you start the first antiretroviral treatment?

Before 2000	☐
2001 to 2010	☐
2011 to 2015	☐
2016 onwards	☐

Do you remember your last CD4 cell count (per ml)?

LESS THAN 200	☐
FROM 200 TO 500	☐
MORE THAN 500	☐
I DO NOT REMEMBER	☐

Do you remember your last plasma HIV-RNA?

NOT DETECTED (undetectable)	☐
DETECTED BETWEEN 50 AND 200 (cp/mL)	☐
DETECTED OVER 200 (cp/mL)	☐
I DO NOT REMEMBER	☐

Which drug do you take for HIV?
Note: a tablet, i.e. a 'drug' with a given commercial name, may contain one or more 'active subtances'.

Two active substances in a tablet *(Dovato®, Juluca®)*	☐
Three active substances in a tablet *(Atripla®, Biktarvy®, Delstrigo®, Eviplera®, Odefsey®, Triumeq®)*	☐
Four active substances in a table *(Genvoya®, Stribild®, Symtuza®)*	☐
Two active substances in two/three tablets	☐
Three/four active substances in two/three tablets-all drugs on the market	☐
I am also taking HIV drugs that are not yet on the market (experimental drugs)	☐
Other	☐
Do not remember	☐

Are you satisfied with yout HIV treatment?

Yes, completely	☐
Yes, but I think it can be improved	☐
No, because I have problems	☐
No, I am not at all	☐
Other / don't know / don't want to answer	☐

Why did your HIV treatment was changed last time??

What I'm taking is my first treatment, I've never changed it.	☐
Because I wasn't well, I couldn't tolerate it (side effects) and I talked to my doctor about it.	☐
Because the biochemical tests (i.e. liver, kidney, cholesterol, triglycerides, etc.) were not going well and the doctor changed it.	☐
Because my doctor suggested another treatment for no particular reason.	☐
Because I was taking too many tablets or because I was taking it several times a day.	☐
Because I changed from three active ingredients (even in one tablet) to two active ingredients (even in one tablet).	☐
Because I switched from two active ingredients (even in one tablet) to three active ingredients (even in one tablet)	☐
Because the viral load had increased and therefore there was an efficacy problem	☐
Other / don't know / don't want to answer	☐

About HIV I talk to…..*(Possible multiple answers)*

All -	☐
My colleagues -	☐
Most friends/friends -	☐
My family -	☐
My partner -	☐

A few (even just one) particular friend(s) -	☐
My doctor/nurse and nobody else - Other	☐
Other / don't know / don't want to answer -	☐

I would….*(Possible multiple answers)*

Having more say in choosing my HIV treatment	☐
To be able to take my HIV treatment not every day, thanks to specially designed medications	☐
Have more frequent dialogue with my infectious diseases doctor	☐
Being more in control of certain medical aspects	☐
Talking more with someone (a professional) not only about the medical aspects of HIV	☐
Know when new drugs are coming and why.	☐
To have a more sincere dialogue with my infectious diseases doctor.	☐
None of the above	☐

3. General Health status

WE NOW ASK YOU FOR SOME DATA ON OTHER ASPECTS OF YOUR HEALTH..

IN THE LAST MONTH, HOW WOULD YOU DESCRIBE YOUR STATE OF HEALTH?

	VERY GOOD	QUITE GOOD	NORMAL	POOR	VERY UNSATISFACTORY
Physical health	☐	☐	☐	☐	☐
Mental health	☐	☐	☐	☐	☐
Sexual health	☐	☐	☐	☐	☐
Overall health	☐	☐	☐	☐	☐

CONSIDERING ALL THE DRUGS OR WHAT YOU TAKE CONTINUOUSLY (whether anti-HIV, for other co-diseases or otherwise), HOW MANY PILLS, BOTTLES, ETC... DO YOU TAKE EACH DAY?

☐ 1 ☐ 2 ☐ 3 ☐ 4 ☐ 5 ☐ 6 ☐ 7 ☐ 8 ☐ 9 ☐ 10 ☐ more than 10

…and how many times per day

☐ 1 ☐ 2 ☐ 3 ☐ 4 ☐ 5 ☐ more than 5

For you….

...HIV is the most important health problem (i.e. disease)	☐
...HIV is not the most important health problem (i.e. pathology)	☐
Don't know/won't answer	☐

I do physical activity/sport ….

Continuously (2 times a week)	☐
2-4 times per month	☐
No, I don't do it	☐
Other / don't know / don't want to answer	☐

I eat….

In a controlled way, healthy food, following a certain diet at least 5 days a week	☐
In a controlled way, healthy food, following a certain diet at least 2 days a week	☐
In a non-controlled way	☐
Other / don’t know / don’t want to answer	☐

I drink alcool….

In a controlled way, in company, a couple of drinks 2 days out of 7 -	☐
In a controlled way, in company, a couple of drinks 4/5 days out of 7 -	☐

In an uncontrolled way -	☐
Other / don't know / don't want to answer	☐

4. HIV treatment burden

TELL US ABOUT YOUR EXPERIENCE WITH ANTI-HIV TREATMENT, CARE AND HIV

MEDICAL INFORMATION ON HIV

Regarding the "reference times" of these questions, please refer, if relevant, to an average of your experiences in the **last 6 months**.

How easy/difficult has it been for you to……

	**Very easy**	**Easy**	**Neither easy nor difficult**	**difficult**	**Very difficult**	**Not applicable**
Get information about your health status from the infectious diseases doctor who is treating you for HIV						
Finding out the information you wanted to know about the HIV drugs you take						
Understand any changes in your treatment plan provided by your HIV infectious disease specialist						
Understand the reasons why you take certain HIV drugs and not others						
Find reliable sources for medical information on various aspects of HIV as well as on HIV medication.						
understand suggestions and/or guidance from your HIV infectious diseases physician						

ANTI-HIV MEDICATIONS

Regarding the "reference times" of these questions, please refer, if relevant, to an average of your experiences in the last month.

HOW MUCH OF A PROBLEM HAS IT BEEN FOR YOU TO…..

	**Not at all**	**A little**	**Somewhat**	**Quite a bit**	**Very much**	**Not applicable**
Organize yourself practically in everyday life with HIV medication (storage, taking, ...)						
Take HIV medication once a day						

Take HIV medication several times a day						
Take HIV medication several times a day at the hospital pharmacy (or locally if so provided in your region)						

'SINGLE-ITEM' INDICATORS OF HIV-MEDICATION BOTHER:
Regarding the "reference times" of these questions, please refer, if relevant, to an average of your experiences in the last month.
HOW BOTHERED HAVE YOU BEEN BY….

	Not at all	A little	Somewhat	Quite a bit	Very much	Not applicable
..how much do you have to rely on your medicine(s)?						
... the side effects of your medicine(s)?						

HIV MEDICAL APPOINTMENTS AND HEALTHCARE SERVICES
Regarding the "reference times" of these questions, please refer, if relevant, to an average of your experiences in the last 6 months.
HOW MUCH OF A PROBLEM HAS IT BEEN FOR YOU TO....

	Not at all	A little	Somewhat	Quite a bit	Very much	Not applicable
...filling in forms (i.e. 'paperwork') for HIV-related health care						
...book your appointments with your HIV infectious disease doctor at times convenient to you						
...make sure that the decisions made with the infectious diseases doctor are then taken on board by other health professionals (e.g. nurses, pharmacists, other medical specialists)						
... wait too long at the hospital pharmacy (or at the local						

pharmacy if this is the case in your region) to pick up your HIV medication.						

INTERPERSONAL CHALLENGES RELATED TO HIV
Regarding the "reference times" of these questions, please refer, if relevant, to an average of your experiences in the last month.
HOW BOTHERED HAVE YOU BEEN BY…

	Not at all	A little	Somewhat	Quite a bit	Very much	Not applicable
...feeling dependent on other people for your HIV-related health needs						
...other people reminding you to do things for your HIV-related health such as taking medicines, scheduling medical appointments...						
...your HIV-related health needs, because they create tension in your relationship with other people						
...other people, because they do not understand your HIV situation.						

ROLE AND SOCIAL ACTIVITY LIMITATIONS
Regarding the "reference times" of these questions, please refer, if relevant, to an average of your experiences in the last month.
HOW MUCH HAS THE HIV, IN YOUR SELF-CARE, INTERFERED WITH YOUR...

	Not at all	A little	Somewhat	Quite a bit	Very much	Not applicable
Work (including working at home)						
Family responsibilities						
Daily activities						
Hobbies and leisure activities						
The ability to spend time with family and/or friends						
The ability to travel for work, holidays, ...						

PHYSICAL AND MENTAL EXHAUSTION HIV-RELATED

Regarding the "reference times" of these questions, please refer, if relevant, to an average of your experiences in the month.

HOW OFTEN THE HIV, IN YOUR SELF-CARE, MAKE YOU FEEL...

	Never	Rarely	Sometimes	Often	Always	Not applicable
Angry						
Preoccupied						
Depressed						
Worn out						
Frustrated						
